# An *Escherichia coli* Expressed Multi-Disulfide Bonded SARS-CoV-2 RBD Shows Native-like Biophysical Properties and Elicits Neutralizing Antisera in a Mouse Model

**DOI:** 10.3390/ijms232415744

**Published:** 2022-12-12

**Authors:** Subbaian Brindha, Takahiro Yoshizue, Rawiwan Wongnak, Hitoshi Takemae, Mami Oba, Tetsuya Mizutani, Yutaka Kuroda

**Affiliations:** 1 Department of Biotechnology and Life Science, Faculty of Engineering, Tokyo University of Agriculture and Technology, 2-24-16 Nakamachi, Koganei-shi 184-8588, Tokyo, Japan; 2 Center for Infectious Disease Epidemiology and Prevention Research, Tokyo University of Agriculture and Technology, 3-5-8 Saiwai-Cho, Fuchu-shi 183-8509, Tokyo, Japan

**Keywords:** disulfide bond, oxidative refolding, subunit antigen, *Escherichia coli* expression, immunogenicity, cell surface markers, pseudovirus neutralization

## Abstract

A large-scale *Escherichia coli* (*E. coli*) production of the receptor-binding domain (RBD) of the SARS-CoV-2 could yield a versatile and low-cost antigen for a subunit vaccine. Appropriately folded antigens can potentially elicit the production of neutralizing antisera providing immune protection against the virus. However, *E. coli* expression using a standard protocol produces RBDs with aberrant disulfide bonds among the RBD’s eight cysteines resulting in the expression of insoluble and non-native RBDs. Here, we evaluate whether *E. coli* expressing RBD can be used as an antigen candidate for a subunit vaccine. The expressed RBD exhibited native-like structural and biophysical properties as demonstrated by analytical RP-HPLC, circular dichroism, fluorescence, and light scattering. In addition, our *E. coli* expressed RBD binds to hACE2, the host cell’s receptor, with a binding constant of 7.9 × 10^−9^ M, as indicated by biolayer interferometry analysis. Our *E. coli*-produced RBD elicited a high IgG titer in Jcl:ICR mice, and the RBD antisera inhibited viral growth, as demonstrated by a pseudovirus-based neutralization assay. Moreover, the increased antibody level was sustained for over 15 weeks after immunization, and a high percentage of effector and central memory T cells were generated. Overall, these results show that *E. coli*-expressed RBDs can elicit the production of neutralizing antisera and could potentially serve as an antigen for developing an anti-SARS-CoV-2 subunit vaccine.

## 1. Introduction

Severe acute respiratory syndrome Coronavirus 2 (SARS-CoV-2) [1], the causative agent of the COVID-19 pandemic, continues to pose a global health threat. There are currently eight approved vaccines [2] against SARS-CoV-2 [1]. Each vaccine modality has advantages and disadvantages. Inactivated and live-attenuated virus-based vaccines are traditional and long-proofed technologies, but they have an inherent risk of incomplete inactivation, resulting in significant safety concerns [3]. In addition, they are not rapidly adaptable to novel strains evading the protection provided by existing vaccines. Even the successful mRNA-based vaccine has drawbacks, requiring deep freeze storage and distribution, which is not easily amenable for low-resource settings [3]. Metal complexes have been proposed as anti-SARS-CoV-2 antibody modifiers, but this technique needs further studies [4,5]. Thus, exploring alternative vaccine modalities is important, as they may overcome some of the disadvantages of current ones. In particular, a subunit vaccine based on *E. coli* expressed proteins could deliver a rapid, cost-effective, and versatile vaccine without the need for sophisticated techniques such as VLP and expression in plant or mammalian cells ([6] and references therein).

SARS-CoV-2 is a single-stranded, positive-sense RNA virus belonging to the Coronaviridae family [7]. SARS-CoV-2 comprises four major structural proteins: the spike (S), envelope, membrane, and nucleocapsid proteins [8]. The S protein forms a homotrimer and mediates viral entry into the host cell by interacting with the host receptor ACE2 (angiotensin-converting enzyme-2 [9,10] through the receptor-binding domain (RBD) [8]. The binding of RBD with ACE2 is crucial for viral infection [11], and RBD is thus a promising candidate for therapeutic drugs and vaccines development [12]. Furthermore, RBD from different SARS-CoV-2 variants has a high degree of sequence identity (97–89%; whereas the full spike protein (S1) sequence identity is 62%) [13], thereby mirroring the possibility of creating polyvalent antibodies [14].

The production of a large amount of well-folded RBD could accelerate the development of vaccine antigens and structural investigations. So far, RBD has been produced in eukaryotic expression systems [15], which is expensive, labor-intensive, and have limited scalability, limiting the therapeutic and clinical development [16]. RBD expressed by *E. coli* could solve these inherent problems [17]. Recently, several *E. coli* expressed proteins have been proposed for use as vaccine antigens. Examples are the hepatitis E vaccine [18], the human papillomavirus vaccine [19], and the meningococcal vaccine [20]. 

RBD is a 25 kDa beta-sheeted protein spanning residues 319–541 of the spike protein [11]. Despite its moderate size, the eight cysteines forming four disulfide bonds in SARS-CoV-2-RBD make its expression in *E. coli* quite challenging, and expression in *E. coli* according to usual protocols produces non-native S-S bonds, resulting in a misfolded protein expressed in the inclusion body. 

Here, we sought to evaluate RBD expressed in *E. coli* as an antigen for a subunit vaccine. In this study, we show that RBD expressed overnight at low temperature in T7 SHuffle cells with vigorous shaking yields an RBD with the native S-S bond pairs exhibiting native-like biophysical properties, as demonstrated by analytical RP-HPLC and spectroscopic methods. In addition, RBD/ACE2 has a binding constant of 7.95 × 10^−9^ nM. Most significantly, RBD elicited a strong immune response with long-term memory in Jcl:ICR mice, and the antisera inhibited the SARS-CoV-2-RBD pseudoviral proliferation, as demonstrated by a pseudovirus-based neutralization assay. In particular, the neutralizing effect suggested that the *E. coli* RBD induces protective immunity in mouse models, and our findings represent a significant advance for the design of a safe and effective SARS-CoV-2 vaccine based on *E. coli*-expressed RBD.

## 2. Results and Discussion

### 2.1. SARS-CoV-2 RBD Expression Using a Bacterial Expression System

SARS-CoV-2 RBD is currently produced in mammalian cells, yeast cells, and baculovirus–insect cells [15,16]. Expression of SARS-CoV-2-RBD in *E. coli* could thus be advantageous from a production cost and scalability viewpoint [21]. However, owing to its four disulfide bonds, *E. coli* expression of SARS-CoV-2-RBD using standard protocols results in insoluble, non-native RBDs with aberrant disulfide bonds [22,23]. The reducing environment of the *E. coli* cytoplasm and the lack of molecular machinery to reshuffle non-native disulfide bonds into native ones make it unsuitable for producing multi-disulfide-bonded proteins [24].

Our strategy was to use an *E. coli* T7 SHuffle strain and express the protein at a low temperature overnight. T7-SHuffle *E. coli* cells are engineered to contain the trxB- and gor-mutations, which alter the redox state for catalyzing the formation of disulfide bonds, and the DsbC (disulfide bond isomerase) gene [25]. T7-SHuffle cells thus enable the oxidative folding of multi-disulfide bonds, and DsbC isomerase can potentially reshuffle non-native SS bonds [26]. Further, lowering the expression temperature to 16 °C slows down protein expression and stabilizes the native structure, thereby aiding the folding process [27]. 

**Figure 1:**
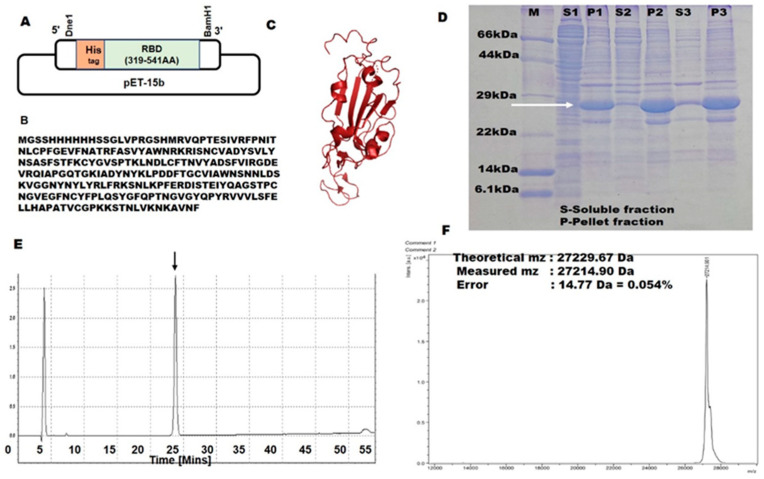
(**A**) Strategy for cloning of SARS-CoV-2 RBD into the pET15b vector with structural demonstration of the SARS-CoV-2-RBD domain. (**B**) amino acid sequence of the target gene cassette. (**C**) Cartoon representation of SARS-CoV-2 RBD generated using Pymol software based on the structure of SARS-CoV-2 RBD (PDB ID-6M0J) [8]. (**D**) Expression analyzed by SDS PAGE. S: a soluble fraction of SARS-CoV-2 RBD, P: pellet fraction of SARS-CoV-2 RBD (1,2,3 represent the rounds of sonication). (**E**) **RP-HPLC purification:** The elution profile of recombinant SARS-CoV-2 RBD shows the presence of a single peak. (**F**) **Molecular weight by MALDI-TOF-MS.** Experimental molecular weight (Exp. Mw) was determined by MALDI-TOF Mass-spectroscopy and calculated molecular weight (Cal. Mw) by Protparam (http://web.expasy.org/protparam/ (accessed on 7 November 2022). Relative errors are in percentages (|Exp. Mw—Cal. Mw|/Cal. Mw × 100).

Large-scale expression of SARS-CoV-2-RBD was carried out using the T7 SHuffle cell line at 16 °C in 200 mL cultures, and the insoluble fractions were solubilized with 6 M Gn HCl. Complete oxidation of the cysteines was achieved by air oxidation for 48 h at pH 8.8 and at 25 °C (with GuHcl). Afterward, the samples were purified by denaturing Ni-NTA chromatography and RP-HPLC. The elution profile of the RP-HPLC showed a single sharp peak (Figure 1E), differing from the fully reduced one by approximately two minutes. The RP-HPLC single peak suggested that all of the protein had folded into a single species of SS bond pairing [28,29,30]. The final yield of SARS-CoV-2-RBD after RP-HPLC was ∼2 mg per 200 mL of LB culture (Figure 1D). The lyophilized RBD was kept at −30 °C until use. 

### 2.2. Structural Integrity and Binding Activity

**Figure 2:**
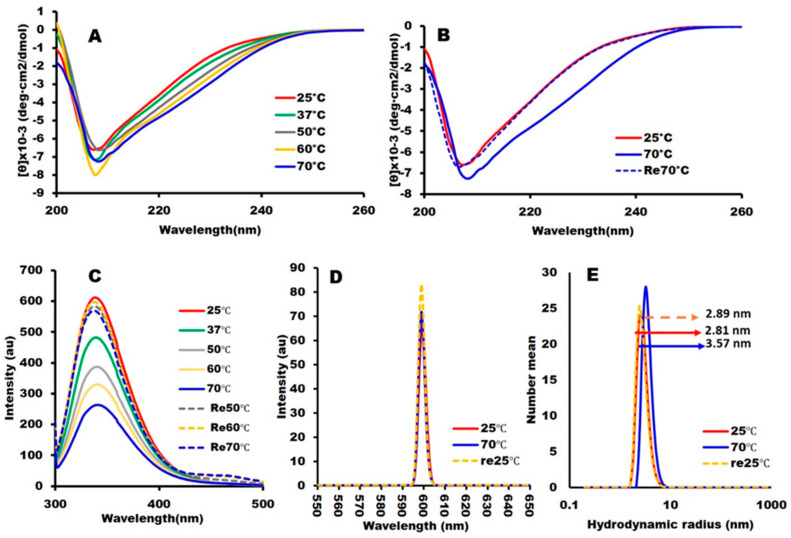
Biophysical and spectroscopic characterization: All spectroscopic measurements were performed at a protein concentration of 0.3 mg/mL in 10 mM Hepes buffer, pH 6.5. **Characterization of the secondary structures:** (**A**) The secondary structures were analyzed using the far-UV CD region (200–260 nm) and (**B**) The reversibility of the CD spectra at 70 °C to 25 °C for SARS-CoV-2-RBD. (**C**) **Characterization of the tertiary structures:** Tryptophan fluorescence intensity (**D**) SARS-CoV-2 RBD particle sizes are measured by dynamic light scattering (DLS) and (**E**) static light scattering (SLS). Line symbols are explained within the panels.

The conformational integrity of the RBD was assessed by circular dichroism (CD) and tryptophan fluorescence spectroscopy. The secondary structure content computed from the far UV-CD spectrum (Figure 2A) using BestSel [31] was 14.0% (19.07%) α-helix and 28.9% (29%) β-sheet, in line with the crystal structure PDB: 6M0J (values in the parentheses indicate the X-ray structure values) [10]. CD spectra measured at temperatures ranging from 25 °C to 70 °C with 10 °C increments and then at 25 °C after cooling indicated cooperative and reversible thermal denaturation (Figure 2A,B), which is a biophysical hallmark for a well-folded protein. The tryptophan fluorescence intensity of SARS-CoV-2-RBD at 25 °C, having an emission maximum of 339 nm (Figure 2C) and exhibiting reversibility from 70 °C, was indicative of the well-folded structure of SARS-CoV-2-RBD. Tryptophan fluorescence at 70 °C indicated a 7 nm redshift (346 nm) and a 2.5-fold decrease in fluorescence intensity (Figure 2C) typical of a change observed during protein unfolding [32,33]. Finally, the SLS intensity of the variant remained low at pH 8.0, indicating the absence of aggregation (Figure 2D), and the hydrodynamic radius (*R*_h_) at pH 8.0w as 2.19 nm, consistent with a monomeric RBD (Figure 2E). Overall, the spectroscopic analysis indicated that our *E. coli* expressed RBD possesses native-like biophysical properties.

**Figure 3:**
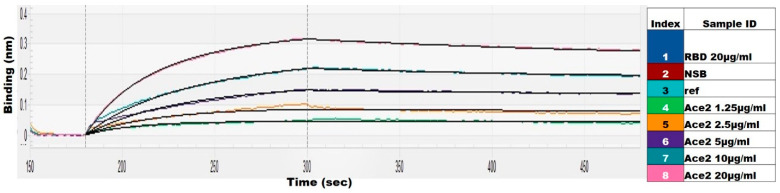
Determination of the binding of recombinant SARS-CoV-2 RBD through the OCTET−N1 Biomolecular Interaction Analyzer. ACE2 in the mobile phase at concentrations of 1.25, 2.5, 5, 10, and 20 µg/m was used.

The binding to the ACE2 (angiotensin-converting enzyme) receptor is a critical step for SARS-CoV-2 to enter target cells [12]. The integrity of the *E. coli*-produced RBD was thus tested by its binding to hACE2 (human ACE2). RBD/hACE2 had a binding affinity of 7.95 × 10^−9^ M (Figure 3), indicating that the *E. coli*-produced RBD recognizes the binding site on ACE2, further confirming its native-like conformation.

### 2.3. Immunogenicity of SARS-CoV-2-RBD in the Mouse Model

**Figure 4:**
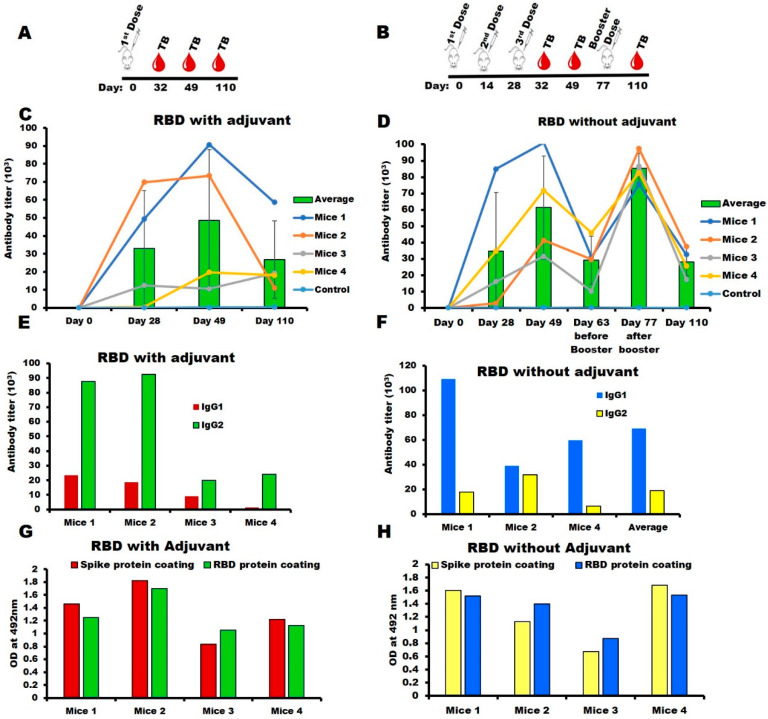
Anti-SARS-CoV-2 RBD sera (IgG) titer assayed by ELISA: IgG detection by ELISA was performed using the tail bleeding (TB) sera. M indicates the mice’s identity; and D indicates the tail bleeding day. Each circle indicates an individual mouse’s titer (total of four mice), and the bars show the average titer. (**A**) Immunization scheme in the presence of adjuvant; (**B**) Immunization scheme in the absence of adjuvant; (**C**) IgG Determination: with adjuvant **and** without adjuvant (**D**); IgG sub-class (IgG1, IgG2a) Determination: with adjuvant (**E**), without adjuvant (**F**); Recognition of mammalian-expressed spike proteins by anti-RBD sera, binding of day 49 serum with mammalian-expressed spike (S1) protein and *E. coli*-expressed RBD as coating antigen for comparison in the adjuvant (**G**) and without adjuvant (**H**) groups (legends are given in the panel).

The titer of the Anti-SARS-CoV-2-RBD antisera produced in Jcl-ICR mice was measured by ELISA using SARS-CoV-2-RBD as the coating antigen. After a single injection of RBD with Titremax adjuvant, the antibody titer increased to over 40,000 on day 28 (Figure 4C). Whereas immunization without adjuvant, three doses were needed to increase the antibody titer (Figure 4D) to levels similar to that of the adjuvant group on day 28. Overall, the high IgG titers in both the adjuvanted and the non-adjuvanted groups showed that the *E. coli*-produced recombinant SARS-CoV-2-RBD was immunogenic. The subclass IgG1 level was much higher than IgG2a in the non-adjuvant group suggesting that SARS-CoV-2-RBD without adjuvant induced a Th2-dependent immune response (Figure 4F), whereas the adjuvant group induced both a Th1- and a Th2-dependent immune response (Figure 4E), which is evident through the presence of higher IgG2a levels than IgG1, indicating that Titermax gold adjuvant shifted the immune response towards the Th1 phenotype. Thus, the choice of adjuvant plays a significant role in the level of involvement of the Th1 and Th2 responses in immunized mice. **Long-term persistence of SARS-CoV-2-RBD-induced immune response in mice:** The anti-SARS-CoV-2-RBD titers produced by the adjuvanted RBD remained high for 15 weeks (Figure 4C,D) indicating a long-term immune response. **Recognition of anti-SARS-CoV-RBD sera with mammalian expressed spike (S1) protein:** SARS-CoV-2-RBD antisera (with and without adjuvant groups) showed strong interaction with the mammalian expressed Spike (S1) protein, essentially identical to the interaction with the *E. coli* expressed SARS-CoV-2-RBD (Figure 4G,H). This confirms the native-like structure of the *E. coli* expressed RBD and antisera raised against the conformational epitopes of the antigen.

### 2.4. Flow Cytometry—Cell Surface Marker Analysis

**Figure 5:**
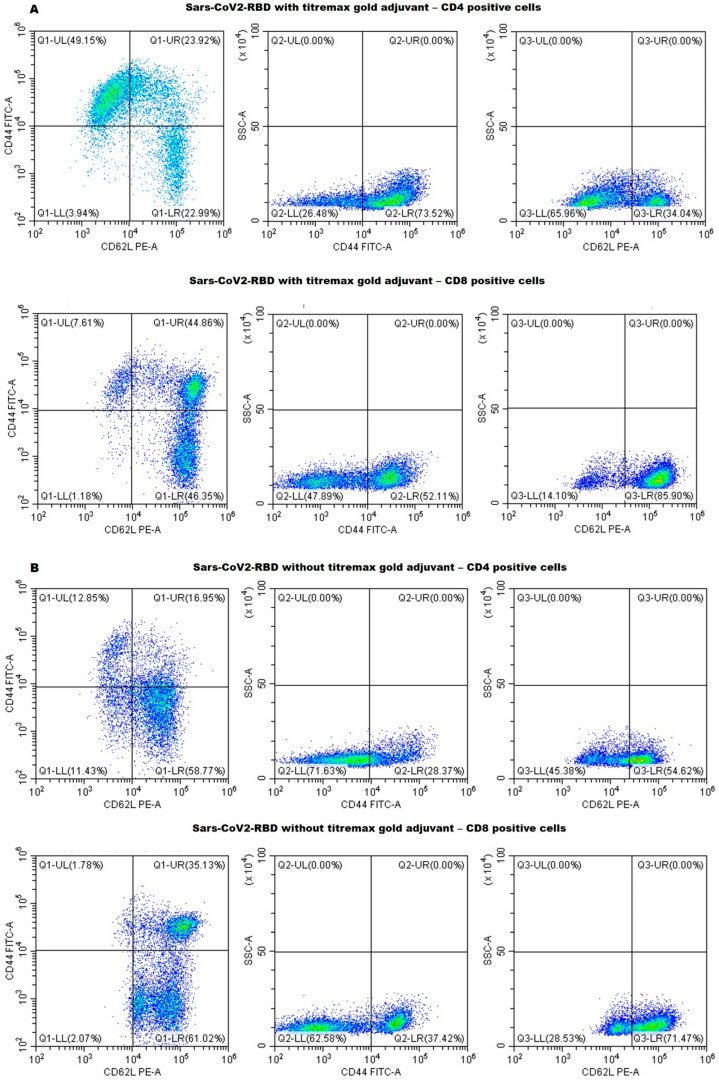
Flow cytometry analysis of cell surface CD (cluster of differentiation) markers. A single-cell suspension of mouse splenocytes in FACS buffer was used for flow-cytometry analysis. Differential expressions of cell surface CD markers induced by T-helper cell (CD4+) and T-cytolytic cell (CD8+) with (**A**) and without (**B**) an adjuvant group. The figure represents data from a single mouse from each group.

**Figure 6:**
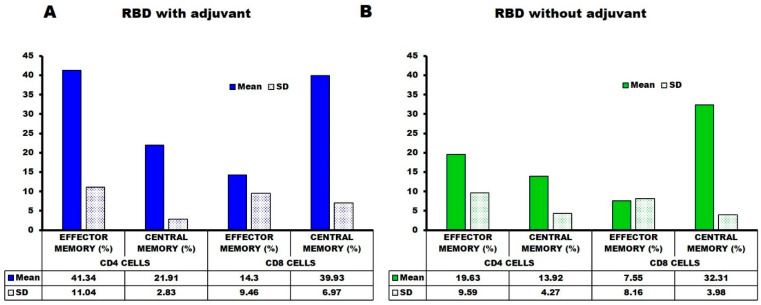
The relative percentages of co-expression of CD44 and CD62L on CD4+ and CD8+ cells are shown in panels, respectively. The results were averaged for two mice and the percentage was indicated as mean ± SD.

The anti-RBD T cell responses and long-term memory were analyzed by CD marker analysis using the spleen collected from mice 18 weeks after the first injection. The mice immunized with SARS-CoV-2-RBD in the presence of Titremax gold adjuvant had a high number of CD44+ and high CD62L+ CD4 (T-helper) and CD8 (T-cytolytic) cells (Figure 5A and Figure 6). A high percentage of CD44+, CD62L+, and CD44+ CD62L-co-expressing T-helper and T-cytolytic cells were observed (Figure 5A and Figure 6), indicating that the SARS-CoV-2-RBD with Titremax gold generated central and effector T-cell memory. On the other hand, the mice immunized with SARS-CoV-2-RBD without Titremax gold also generated central and effector T-cell memory but had fewer CD44+ CD62L+ and CD44+ CD62L- co-expressing T-helper (12.85%) and T-cytolytic (1.78%) cells in comparison with the adjuvant group (Figure 5A and Figure 6). These observations provide evidence that *E. coli*-produced RBD can establish a long-term, target-specific immune response in a way necessary for the development of a protein-based vaccine.

### 2.5. E. coli-Produced SARS-CoV-2-RBD Can Induce Specific and Neutralizing Antibodies

**Figure 7:**
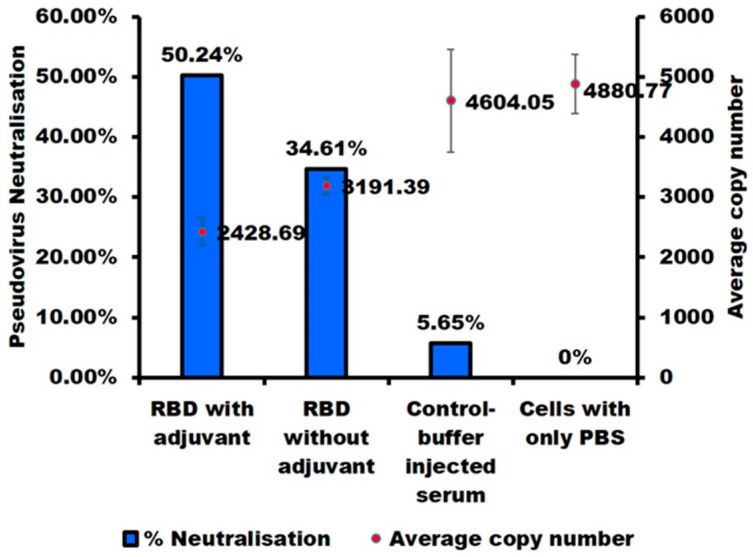
SARS-CoV-2 S pseudotyped virus neutralization assay. Vero E6/TMPRSS2 cells were transduced with a SARS-CoV-2 S (D614G) pseudotyped lentivirus in the presence of mouse sera. 24 h later, cells were collected, and RNA was extracted. The copy number of the S gene (codon-optimized) was examined for determining the percentage of neutralization. Dots represent the mean values of gene copy number, and the bar represents the % inhibition of three technical replicates.

A decisive step for identifying an antigen as a vaccine candidate is its ability to produce neutralizing antibodies. We thus assessed the neutralizing activity of the anti-RBD antisera produced with and without adjuvant using SARS-CoV-2 wild-type (D614G) pseudoviruses. The antisera neutralized SARS-CoV-2 pseudovirus with an efficacy of 51% for the 110th-day adjuvanted antisera and 35% for the non-adjuvanted antisera (Figure 7). These results further confirm that the *E. coli*-produced RBD harbors native-like epitopes and are able to produce the neutralizing antibodies. Noteworthy, though the neutralization ability of RBD expressed in mammalian, insect, and yeast cells have previously been reported [7,10,15,16], this is the first such report for an RBD produced in *E. coli* without a fusion protein.

## 3. Materials and Methods

### 3.1. Protein Expression and Purification

A synthetic gene encoding RBD (UniProt ID P0DTC2) was designed with codon optimization for *E. coli*. The cysteine at the 538th amino acid position was replaced with an alanine. The gene fragment was subcloned in the pET15b vector between Nde1 and BamH1 restriction site, yielding the expression vector pET15b-poly (His)6-RBD.

The recombinant plasmid was transformed into *E. coli* T7 Shuffle cells (New England Biolabs, Ipswich, MA, USA). The cells containing recombinant plasmids were pre-cultured in 5 mL of LB containing 50 μg/mL ampicillin and incubated overnight at 30 °C with shaking at 220 rpm. Then, 5 mL of cell pre-culture was transferred into a 200 mL LB medium with appropriate antibiotics. The cell cultures were incubated at 30 °C with shaking until the cell density reached OD600 ≈ 0.6. Gene expression was induced by the addition of 0.25 mM IPTG and thereby incubating with 220 rpm shaking at 16 °C for 16–18 h, and cells were harvested by centrifugation at 8000 rpm (9422× *g*; Hitachi himac CF16RX centrifuge with a T9A31 rotor) (Hitachi, Tokyo, Japan) at 4 °C for 20 min. The cell pellets were resuspended in lysis buffer (50 mM Tris-HCl pH 8, 150 mM NaCl) and disrupted by sonication in buffer (50 mM Tris-HCl pH 8, 1% NP-40 (*v*/*v*), 0.1% Deoxycholic acid (*w*/*v*) and 5 mM EDTA), and inclusion bodies were obtained after centrifugation at 8000 rpm for 20 min. The protein expression was analyzed by SDS gel electrophoresis.

RBD is expressed in the inclusion body. First, inclusion bodies were solubilized and air oxidized for 48 h at 25 °C in 6 M guanidine hydrochloride with 50 mM Tris–HCl, pH 8.7. After centrifugation at 8000 rpm at 4 °C for 20 min and filtration through a 0.2 µm filter, the 6 × histidine-tagged protein was purified by using denaturing open nickel-nitrilotriacetic acid (Ni-NTA) (Wako, Tokyo, Japan) chromatography. The column was washed three times with the wash buffer (6 M GuHCl, 50 mM Tris-HCl pH 6.8), and proteins were eluted with the elution buffer (6 M GuHCl and 10% Acetic Acid). GuHcl was removed by 18 h dialysis against reverse osmosis (RO) water (four times exchange of the outer solution) at 4 °C (The molecular weight cut-off [MWCO] of the dialysis membrane was 14,000). The sample was then centrifuged at 8000 rpm, 4 °C for 20 min, and the supernatant was separated from the debris. 

The proteins were further purified by reverse-phase (RP) high-performance liquid chromatography (HPLC; Shimadzu, Kyoto, Japan) using an Intrada 5WP-RP column (Imtakt, Kyoto, Japan), and absorbance at 220 nm was used to monitor the HPLC runs. Solution A (MilliQ-water + 0.1% trifluoroacetic acid (TFA)) and Solution B (Acetonitrile + 0.05% TFA) were used as mobile phases with a flow rate of 1 mL/min and a column temperature of 30 °C. The analytical RP-HPLC purification was performed using acetic acid at a final concentration of 10% (*v*/*v*) and filtered with a 0.20 µm membrane filter to remove any aggregates. The purified RBD (purity: >95%) were lyophilized and stored at −30 °C.

### 3.2. Physicochemical Measurements

We measured the biophysical properties of the proteins in 10 mM Hepes buffer, pH 6.5, at a protein concentration of 0.3 mg/mL, which is the concentration we used for the immunogenic studies. Stock solutions were prepared by dissolving the protein in MQ. The stock solutions were centrifuged at 20,000× *g* for 20 min at 4 °C and filtered with a 0.20 μm membrane filter (MDI Membrane Technology, Ambala Cantt, India) to remove any aggregates. The final samples were prepared by diluting the stock solutions to a final protein concentration of 0.3 mg/mL in 10 mM Hepes buffer (pH 6.5). Far-UV circular dichroism (CD): CD was measured using a 2 mm path-length quartz cuvette in a continuous scanning mode. For each measurement, three scans were accumulated from 200 nm to 260 nm wavelength at temperatures ranging from 25 °C to 70 °C with 10 °C increments. Reversibility was assessed by measuring the spectrum after cooling the sample to 25 °C. The secondary structure content was calculated using BeStSel [22]. Tryptophan fluorescence: Trp fluorescence spectra with λex at 295 nm, respectively, were measured on an FP-8500 spectrofluorometer (JASCO, Tokyo, Japan) using a quartz cuvette with a 3 mm optical path length. The sample’s temperature increased from 25 °C to 70 °C and decreased back to 25 °C to assess reversibility. Light scattering: Dynamic light scattering (DLS) measurements were performed at 25 °C, 70 °C, and reverse 25 °C for pH 8.0, using a polystyrene cuvette with a Zeta-nanosizer (Malvern, UK). The hydrodynamic radius [*R*_h_] was determined from the size distribution using the Stokes-Einstein equation and averaged over three individual measurements. Static light scattering (SLS) measurements were performed under the same conditions at a wavelength of 600 nm with an FP-8500 spectrofluorometer (JASCO, Tokyo, Japan) using a quartz micro-cuvette with an optical path length of 3 mm.

The RBD binding affinity with hACE2 (purity > 90% SDS-PAGE) (Bioworld tech, St. Louis Park, MN, USA) was measured using an OCTET-N1 Biomolecular Interaction Analyzer (Satorius, Goettingen, Germany). A 5 μg/mL concentration of SARS-CoV-2 RBD was immobilized on the biosensor surface for 300 s. The baseline interference phase was obtained by taking measurements for 60 s in a kinetics buffer (KB: 1X Hepes and 0.02% Tween-20), and then the sensors were subjected to association phase immersion for 400 s in wells containing recombinant hACE2 diluted in HEPES buffer. Then, the sensors were immersed in KB for as long as 400 s in the dissociation step. The RBD binding affinities for ACE2 were calculated from global fitting with a 1:1 Langmuir binding model.

### 3.3. Mice Immunization

Antigen formulation: two sets of immunization experiments were carried out with Jcl:ICR (CLEA, Shizuoka, Japan) mice, all aged four weeks at the start of the experiment. One group (number of mice = four) was carried out without adjuvant, and the other group (number of mice = four) was carried out in the presence of a Titremax Gold adjuvant (Sigma-Aldrich, St. Louis, MO, USA). Immunization in the presence of adjuvant: The RBD were dissolved in 10 mM Hepes buffer, pH 7.4 at 30 µg/dose and supplemented with an equal volume of Titermax Gold adjuvant (100 µL protein plus 100 µL adjuvant; for a total of 200 µL/dose/mice). The first dose was given subcutaneously, and after that, no more doses were administered. Immunization in the absence of adjuvant: RBD was formulated in Hepes, pH 7.4 at 30 µg/dose (100 µL/mice) and injected subcutaneously at biweekly intervals unless otherwise specified. In addition, one negative control mouse was injected with only hepes buffer, and other mice were injected with an equal volume of hepes and Titermax Gold adjuvant. Specific immune responses (anti-RBD IgG) were monitored on days 28, 49, 77, and 110 after inoculation using tail-bleed samples (of all mice in each group) through ELISA. After monitoring, all mice were euthanized, blood samples were collected from the heart and centrifuged, and the sera were preserved at −30 °C in aliquots of 50 µL until use. Long-term immunogenicity: The long-term immune response was monitored by measuring the serum antibody titers for up to 110 days (15 weeks) after the immunization. All of the experiments were performed in compliance with the Tokyo University of Agriculture and Technology and Japanese government regulations on animal experimentation.

Measurement of Serum IgG Level: The antisera’s IgG, IgG1, and IgG2a levels were assessed individually by ELISA using RBD as a coating antigen. SARS-CoV-2 antisera were applied to the 96-well ELISA plates at an initial dilution of 1:100 for tail-bleed samples. Plates were then incubated at 37 °C for 2 h. After washing the plates thrice with PBS-0.05% Tween-20, each well was filled with 100 μL of anti-mouse IgG HRP conjugate (Thermo Fisher Scientific, Waltham, MA, USA) at a 1:3000 dilution (or its subclasses: IgG1, 1:25,000 dilution and IgG2a, 1:3000 dilution) in 0.1% BSA-PBS-Tween-20 and incubated at 37 °C for 1.5 h. As a substrate, OPD (o-phenylenediamine dihydrochloride) was added. Color intensities were measured at 492 nm using a microplate reader (SH9000 Lab, Hitachi High-Tech Science Co., Tokyo, Japan) immediately after stopping the reaction with 1 N sulfuric acid (50 µL/well). Finally, titers were calculated using a power-fitting model, and values were averaged over the number of mice (n).

Furthermore, the binding of RBD antisera to full-length spike protein (S1) expressed in mammalian cells, purity > 95% (SDS-PAGE) (Invivogen, San Diego, CA, USA), was tested by ELISA. The plate was coated separately with 100 µL of 2.5 µg/mL of the mammalian expressed full-length spike S1 protein in 1X PBS (pH 7.4), and the following steps are mentioned above. 

All of the experiments were performed in compliance with TUAT’s regulations and Japanese governmental guidance on animal experimentation.

### 3.4. Cell Surface CD Marker Analysis

Cell surface CD markers on splenocytes extracted on the 147th day after the last dose were investigated by flow cytometry. Experimental samples (single mice from each group) were prepared according to our reported protocol [21,22,23,24]. In short, single-cell suspensions of mice splenocytes were prepared in a FACS (Fluorescence Activated cell Sorter) buffer (PBS supplemented with 2% FBS (Fetal Bovine Serum), 1 mM EDTA, and 0.1% sodium azide). Afterward, 1X RBC lysis buffer (0.15 M ammonium chloride, 10 mM potassium bicarbonate, and 0.1 mM EDTA) was used to lyse the red blood cells (RBCs). Furthermore, 1 million splenocyte cells in 100 µL pre-cooled FACS buffer were surface stained with different fluorescent-labeled antibodies according to the manufacturer’s guidelines. To study the CD4 T-lymphocytes, cells were stained with anti-CD3-Pcy5, CD4-Pcy7, CD44-FITC, and CD62LPE-conjugated antibodies in one tube, and for CD8 T-lymphocytes, cells were stained with anti-CD3-Pcy5, CD8-Pcy7, CD44-FITC, and CD62LPE-conjugated antibodies in another tube (0.2 μg of antibodies/100 μL) for 30 min in the dark. Unbound excess conjugated antibodies were removed by centrifugation, and the cells were resuspended in 500 μL of FACS buffer. The data were collected using a CytoFlex flowcytometer (Beckman Coulter, Brea, CA, USA). 

### 3.5. Pseudovirus-Based Neutralization Assay

Antisera were collected to evaluate the neutralizing efficacy on days 49, 70, and 110. Antisera were heated at 56 °C for 30 min before use. Neutralizing antibodies were detected using pseudoviruses. The SARS-CoV-2-variant pseudovirus was prepared according to the protocol of the Lenti-Pac SARS-CoV-2 full-length spike protein-pseudotyped (D614G) Lentivirus Packaging Kit (GeneCopoeia, Rockville, MD, USA. Briefly, 293T cells (2.5 × 10^5^ cells) were seeded onto a 6-well culture plate in DMEM/5%FBS 2 days before transfection. Cells were then transfected with 1 mg of Pseudotype packaging mix using EndoFectin Lenti transfection reagent. At 16 h post-transfection, the culture medium was replaced with 2 mL of DMEM/2%FBS, and TiterBoost Reagent was added to the medium. At 48 hr post-transfection, the supernatant containing the SARS-CoV-2-variant (D614G) pseudovirus was collected and stored at −80 °C until use. For the neutralization assay, Vero E6/TMPRSS2 cells were seeded onto a 24-well culture plate in EMEM/5%FBS 24 h before the assay. The D614G pseudovirus (135 μL) was mixed with 15 μL of heat-inactivated mouse serum and incubated at 37 °C for 1 h. The virus-serum mixture was added to the cells in EMEM/5% FBS containing polybrene (final: 10 μg/mL). After 24-h incubation at 37 °C in a 5% CO_2_ incubator, the cells from each well were harvested separately, and RNA isolation was performed using a Qiagen RNAeasy kit (Hilden, Germany) according to the manufacturer’s protocol. For the qPCR assay, one hundred ng of total RNA were amplified using the One Step PrimeScript RT-PCR Kit (TaKaRa Bio, Otsu, Japan) as described before [34]. Primers and probes were designed from the codon-optimized S gene sequence of the D614G pseudovirus. The sequences were as follows: primer F: 5′-GCAAGATCGCCGACTACAA-5′, primer R: 5′-TTGCCTCCAACTTTCGAATCTA-3′, probe: 5′-56-FAM/TACAAGCTG/ZEN/CCTGACGACTTCACC/3IABkFQ/-3′. The thermal cycling condition was 45 °C for 5 min and 95 °C for 30 s, followed by 40 cycles of 95 °C for 10 s, 55 °C for 20 s, and 72 °C for 20 s. Fluorescence signal data were analyzed using an automatic quantification algorithm in LightCycler Nano Software 1.1 (Roche Diagnostics, Manheim, Germany). 

## 4. Conclusions

*E. coli*–expressed RBD can be prepared in ten days or less and thus could thus offer a cost efficient and versatile subunit vaccine antigen. Issues as refolding and low-immunogenicity need to be overcome, but our *E. coli*-produced SARS-CoV-2-RBD protein. which exhibited native-like biophysical and structural properties, triggered the production of neutralizing antibodies in Jcl:ICR mice. We believe the present results represent a significant advance toward using *E. coli*-produced proteins as subunit vaccine antigens.

## Data Availability

Not applicable.

## References

[1] Coronaviridae Study Group of the International Committee on Taxonomy of Viruses (2020). The species Severe acute respiratory syndrome-related coronavirus: Classifying 2019-nCoV and naming it SARS-CoV-2. Nat. Microbiol..

[2] Mahase E. (2020). COVID-19: What do we know about the late stage vaccine candidates?. Brit. Med. J..

[3] Zhang N., Jiang S., Du L. (2014). Current advancements and potential strategies in the development of MERS-CoV vaccines. Expert Rev. Vaccines.

[4] Abd El-Lateef H.M., El-Dabea T., Khalaf M.M., Abu-Dief A.M. (2022). Development of Metal Complexes for Treatment of Coronaviruses. Int. J. Mol. Sci..

[5] Al-Radadi N.S., Abu-Dief A.M. (2022). Silver nanoparticles (AgNPs) as a metal nano-therapy: Possible mechanisms of antiviral action against COVID-19. Inorg. Nano-Met. Chem..

[6] Luo F., Liao F.L., Wang H., Tang H.B., Yang Z.Q., Hou W. (2018). Evaluation of antibody-dependent enhancement of SARS-CoV infection in rhesus macaques immunized with an inactivated SARS-CoV vaccine. Virol. Sin..

[7] Salzer R., Clark J.J., Vaysburd M., Chang V.T., Abecka A.A., Kiss L., Sharma P., Llamazares A.G., Kipar A., Hiscox J.A. (2021). Single-dose immunisation with a multimerised SARS-CoV-2 receptor binding domain (RBD) induces an enhanced and protective response in mice. FEBS Lett..

[8] Naqvi A.A.T., Fatima K., Mohammad T., Fatima U., Singh I.K., Singh A., Atif S.M., Hariprasad G., Hasan G.M., Hassan M.I. (2020). Insights into SARS-CoV-2 genome, structure, evolution, pathogenesis and therapies: Structural genomics approach. Biochim. Biophys. Acta Mol. Basis Dis..

[9] V’kovski P., Kratzel A., Steiner S., Stalder H., Thiel V. (2021). Coronavirus biology and replication: Implications for SARS-CoV-2. Nat. Rev. Microbiol..

[10] Lan J., Ge J., Yu J., Shan S., Zhou H., Fan S., Zhang Q., Shi X., Wang Q., Zhang L. (2020). Structure of the SARS-CoV-2 spike receptor-binding domain bound to the ACE2 receptor. Nature.

[11] Li W., Moore M.J., Vasilieva N.J., Sui J., Wong S.K., Berne M.A., Somasundaran M., Sullivan J.L., Luzuriaga K., Greenough T.C. (2003). Angiotensin-converting enzyme 2 is a functional receptor for the SARS coronavirus. Nature.

[12] Yun C., Yao G., Yihang P., Zhizhuang J.Z. (2020). Structure analysis of the receptor binding of 2019-nCoV. Biochem. Biophys. Res. Commun..

[13] Wanbo T., Lei H., Xiujuan Z., Jing P., Denis V., Shibo J., Yusen Z., Lanying D. (2020). Characterization of the receptor-binding domain (RBD) of 2019 novel coronavirus: Implication for development of RBD protein as a viral attachment inhibitor and vaccine. Cell. Mol. Immunol..

[14] Jaimes J.A., André N.M., Chappie J.S., Millet J.K., Whittaker G.R. (2020). Phylogenetic Analysis and Structural Modeling of SARS-CoV-2 Spike Protein Reveals an Evolutionary Distinct and Proteolytically Sensitive Activation Loop. J. Mol. Biol..

[15] Saunders K.O., Lee E., Parks R., Martinez D.R., Li D., Chen H., Edwards R.J., Gobeil S., Barr M., Mansouri K. (2021). Neutralizing antibody vaccine for pandemic and pre-emergent coronaviruses. Nature.

[16] Arbeitman C.R., Auge G., Blaustein M., Bredeston L., Corapi E.S., Craig P.O., Cossio L.A., Dain L., D’Alessio C., Elias F. (2020). Argentinian AntiCovid Consortium Structural and functional comparison of SARS-CoV-2-spike receptor binding domain produced in *Pichia pastoris* and mammalian cells. Sci. Rep..

[17] Müller D., Bayer K., Mattanovich D. (2006). Potentials and limitations of prokaryotic and eukaryotic expression systems for recombinant protein production—A comparative view. Microb. Cell Fact..

[18] Proffitt A. (2012). First HEV vaccine approved. Nat. Biotechnol..

[19] Qiao Y.L., Wu T., Li R.C., Hu Y.M., Wei L.H., Li C.G., Chen W., Huang S., Zhao F., Li M. (2020). Efficacy, safety, and immunogenicity of an Escherichia coli-produced bivalent human papillomavirus vaccine: An interim analysis of a randomized clinical trial. J. Natl. Cancer Inst..

[20] Shirley M., Taha M.K. (2018). MenB-FHbp meningococcal group B vaccine (Trumenba): A review in active immunization in individuals aged ≥ 10 years. Drugs.

[21] Rosano G.L., Ceccarelli E.A. (2014). Recombinant protein expression in *Escherichia coli*: Advances and challenges. Front Microbiol..

[22] Tantiwiwat T., Thaiprayoon A., Siriatcharanon A.K., Tachaapaikoon C., Plongthongkum N., Waraho-Zhmayev D. (2022). Utilization of Receptor-Binding Domain of SARS-CoV-2 Spike Protein Expressed in *Escherichia coli* for the Development of Neutralizing Antibody Assay. Mol. Biotechnol..

[23] Salem R., El-Kholy A.A., Waly F.R., Ayman D., Sakr A., Hussein M. (2021). Generation and utility of a single-chain fragment variable monoclonal antibody platform against a baculovirus expressed recombinant receptor binding domain of SARS-CoV-2 spike protein. Mol. Immunol..

[24] Missiakas D., Georgopoulos C., Raina S. (1994). The Escherichia coli dsbC (xprA) gene encodes a periplasmic protein involved in disulfide bond formation. EMBO J..

[25] Lobstein J., Emrich C.A., Jeans C., Faulkner M., Riggs P., Berkmen M. (2012). SHuffle, a novel Escherichia coli protein expression strain capable of correctly folding disulfide bonded proteins in its cytoplasm. Microb. Cell Factories.

[26] Hartl F.U., Hartl M.M. (2009). Converging concepts of protein folding in vitro and in vivo. Nat. Struct. Mol. Biol..

[27] Chen X., Li C., Liu H. (2021). Enhanced recombinant protein production under special environmental stress. Front. Microbiol..

[28] Veldkamp C.T., Koplinski C.A., Jensen D.R., Peterson F.C., Smits K.M., Smith B.L., Johnson S.K., Lettieri C., Buchholz W.G., Solheim J.C. (2016). Production of Recombinant Chemokines and Validation of Refolding. Methods Enzymol..

[29] Brindha S., Kibria M.G., Saotome T., Unzai S., Kuroda Y. (2021). EGFR extracellular domain III expressed in Escherichia coli with SEP tag shows improved biophysical and functional properties and generate anti-sera inhibiting cancer cell growth. Biochem. Biophys. Res. Commun..

[30] Brindha S., Kuroda Y. (2022). A Multi-Disulfide Receptor-Binding Domain (RBD) of the SARS-CoV-2 Spike Protein Expressed in *E. coli* Using a SEP-Tag Produces Antisera Interacting with the Mammalian Cell Expressed Spike (S1) Protein. Int. J. Mol. Sci..

[31] Micsonai A., Wien F., Bulyáki É., Kun J., Moussong É., Lee Y.H., Goto Y., Réfrégiers M., Kardos J. (2018). BeStSel: A web server for accurate protein secondary structure prediction and fold recognition from the circular dichroism spectra. Nucleic Acids Res..

[32] Lehrer S.S. (1971). Solute perturbation of protein fluorescence. Quenching of the tryptophyl fluorescence of model compounds and of lysozyme by iodide ion. Biochemistry.

[33] Vivian J.T., Callis P.R. (2001). Mechanisms of tryptophan fluorescence shifts in proteins. Biophys. J..

[34] Tsuchiaka S., Masuda T., Sugimura S., Kobayashi S., Komatsu N., Nagai M., Omatsu T., Furuya T., Oba M., Katayama Y. (2016). Development of a novel detection system for microbes from bovine diarrhea by real-time PCR. J. Vet. Med. Sci..

